# Retrospective imaging studies of gastric cancer: Study protocol clinical trial (SPIRIT Compliant)

**DOI:** 10.1097/MD.0000000000019157

**Published:** 2020-02-21

**Authors:** Zixing Huang, Dan Liu, Xinzu Chen, Pengxin Yu, Jiangfen Wu, Bin Song, Jiankun Hu, Bing Wu

**Affiliations:** a Department of Radiology, West China Hospital; b Department of Gastrointestinal Surgery and Laboratory of Gastric Cancer, West China Hospital, Sichuan University, Chengdu; c Institute of Advanced Research, Infervision, Beijing, China.

**Keywords:** computed tomography, deep learning, gastric cancer, neural networks, peritoneal metastasis, X-ray

## Abstract

**Introduction:**

Peritoneal metastasis (PM) is a frequent condition in patients presenting with gastric cancer, especially in younger patients with advanced tumor stages. Computer tomography (CT) is the most common noninvasive modality for preoperative staging in gastric cancer. However, the challenges of limited CT soft tissue contrast result in poor CT depiction of small peritoneal tumors. The sensitivity for detecting PM remains low. About 16% of PM are undetected. Deep learning belongs to the category of artificial intelligence and has demonstrated amazing results in medical image analyses. So far, there has been no deep learning study based on CT images for the diagnosis of PM in gastric cancer.

**We proposed a hypothesis:**

CT images in the primary tumor region of gastric cancer had valuable information that could predict occult PM of gastric cancer, which could be extracted effectively through deep learning.

**Objective:**

To develop a deep learning model for accurate preoperative diagnosis of PM in gastric cancer.

**Method:**

All patients with gastric cancer were retrospectively enrolled. All patients were initially diagnosed as PM negative by CT and later confirmed as positive through surgery or laparoscopy. The dataset was randomly split into training cohort (70% of all patients) and testing cohort (30% of all patients). To develop deep convolutional neural network (DCNN) models with high generalizability, 5-fold cross-validation and model ensemble were utilized. The area under the receiver operating characteristic curve, sensitivity and specificity were used to evaluate DCNN models on the testing cohort.

**Discussion:**

This study will help us know whether deep learning can improve the performance of CT in diagnosing PM in gastric cancer.

## 1 Introduction

Although it is steadily declining in incidence, gastric cancer (GC) remains one of the most common and deadly tumor in the world. According to GLOBOCAN 2018 data, GC is the 5th most common and the 3rd most deadly cancer.^[1]^ Peritoneal metastasis (PM) is a frequent condition in patients presenting with GC, especially in younger patients with advanced tumor stages.^[2]^ Given the detrimental influence of PC on survival, efforts should be undertaken to further explore the promising results that were obtained in preventing or treating this condition with multimodality strategies.^[2]^ Therefore, detecting noninvasively PM of GC before surgery would be crucial for avoiding unnecessary resection and selecting optimal therapy in clinical practice.^[3–8]^

CT is the most common noninvasive modality for preoperative staging in GC.^[4,6,8–10]^ Omentum cake, large amounts of ascites, and parietal peritoneal thickening are typical CT features of PM.^[11]^ However, the challenges of limited CT soft tissue contrast result in poor CT depiction of small peritoneal tumors, which are often indistinguishable from adjacent bowel, mesentery, and mucin.^[12–14]^ These features were not present in all patients with PM, the sensitivity for detecting PM remains low.^[11,15–17]^ About 16% of PM are undetected.^[18]^ PM-negative of patients with GC diagnosed by preoperative CT were confirmed with PM during subsequent surgeries or laparoscopies, which is known as occult PM.^[11,19]^ At present, laparoscopy is the most reliable preoperative method to identify clinically occult PM in patients with GC.^[4,7–9,12,20]^ However, laparoscopy is an invasive and costly procedure and is not systematic in clinical practice. Therefore, we should develop better noninvasive methods to diagnose PM beyond conventional imaging.

There are 2 kinds of medical image analysis method that have entered the stage of rapid development: radiomics and deep learning.^[21]^ Recently, several studies have shown that radiomics based on CT can help diagnose PM in GC.^[22–24]^ Deep learning belongs to the category of artificial intelligence and has demonstrated amazing results in medical image analyses, such as detecting diabetic retinopathy in fundus photographs,^[25]^ classifying skin cancer from skin photographs,^[26]^ and detecting metastasis on pathologic images,^[27]^ and so on. So far, there has been no deep learning study based on CT images for the diagnosis of PM in GC.

We proposed a hypothesis: CT images in the primary tumor region of GC had valuable information that could predict occult PM of GC, which could be extracted effectively through deep learning.

## 2 Participants and methods

### 2.1 Study aims

The aim of this study is to develop a deep learning model for accurate preoperative diagnosis of PM in GC.

### 2.2 Study design/setting

This is a single-center, retrospective, cross-sectional clinical study. The CT data from the Surgical Gastric Cancer Patient Registry of West China Hospital (id: WCH-SGCPR-2019-08), all of which were anonymous.

### 2.3 Study registration

This clinical trial has been registered on the Chinese Clinical Trial Registry (www.chictr.org.cn), and the registration number is ChiCTR1900028437.

### 2.4 Eligibility criteria (Fig. 1)

The patients with GC underwent surgery or laparoscopic exploration at our hospital from January 2013 to December 2016 were retrospectively collected.

**Figure 1:**
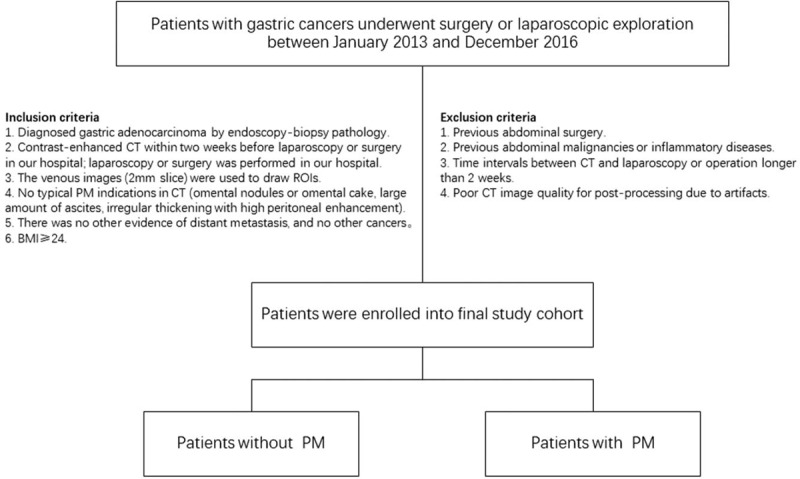
Flowchart of the patient selection and patient exclusion.

#### 2.4.1 Inclusion criteria

(1) Diagnosed gastric adenocarcinoma by endoscopy-biopsy pathology.
(2) Contrast-enhanced CT within 2 weeks before laparoscopy or surgery in our hospital; laparoscopy or surgery was performed in our hospital.
(3) The venous images (2 mm slice) were used to draw region of interest (ROIs).
(4) No typical PM indications in CT (omental nodules or omental cake, large amount of ascites, irregular thickening with high peritoneal enhancement).
(5) There was no other evidence of distant metastasis, and no other cancers.
(6) Body mass index ≥24.

#### 2.4.2 Exclusion criteria

(1) Previous abdominal surgery.
(2) Previous abdominal malignancies or inflammatory diseases.
(3) Time intervals between CT and laparoscopy or operation longer than 2 weeks.
(4) Poor CT image quality for post-processing due to artifacts.

### 2.5 Intervention

This study is a retrospective analysis of CT images and deep learning modeling, with no intervention measures for patients.

### 2.6 Deep learning methods

#### 2.6.1 CT image acquisition and preprocessing

All patients underwent enhanced CT examination within 2 weeks before surgery or laparoscopic exploration.

Portal vein phase CT images were exported to the ITK-SNAP software (version 2.2.0; www.itksnap.org) for manual segmentation. The lesions of GCs were manually recognized by a radiologist with 5 years’ experience in gastroenterology imaging) and confirmed by another abdominal radiologist with 14 years’ experience in gastroenterology imaging). Two radiologists reviewed all slices of a patient and selected 1 slice with the largest tumor area and manually drawn along the margin of the lesion. The gastric lumen and artifacts were carefully avoided. The images with ROIs derived from ITK-SNAP as input were used to construct the deep learning-based model.

#### 2.6.2 Data management

For the development of the deep learning-based model, all patients’ CT images with PM-negative and patients’ CT images with occult PM, were randomly allocated into 1f of 2 cohorts:

(1) the training data cohort contains 70% patients of occult PM and 70% patients of PM-negative, and 5-fold cross-validation was used to optimize the network weight;
(2) the in-house validation data cohort comprising 30% patients of occult PM and 30% patients of PM-negative to evaluate the predictive performance of the deep learning-based model.

#### 2.6.3 Development of the deep learning-based model

In brief, we adopted a deep convolutional neural network with Depthwise Convolution blocks and Fully Connection layer constructing a classifier. This classifier was designed to determine the probability of a patient having an occult PM. For training the model can predict occult PM, we used weighted cross-entropy loss to supervise the model's parameter optimization direction.

**Figure 2:**
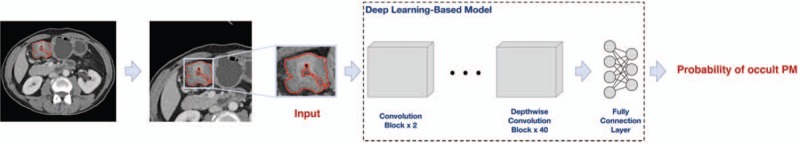
Deep learning workflow in this study.

Finally, for each input of CT image, the deep learning-based model provided a continuous value between 0 and 1 as the probability of occult PM, and the process is shown in Figure 2.

#### 2.6.4 Evaluation of the deep learning-based model

We get 5 different single models via 5-fold cross-validation. Then, we carry out all 5 models as a vanilla ensemble model, averaging the predicted risk values of the 5 single models. We analyzed the ROC consistency between the Vanilla ensemble model and each single model. Moreover, we selected each single model as the candidate model based on the above analysis results, and then assembled these to obtain our ensemble model. We evaluate the performance of models on the in-house validation cohort.

### 2.7 Outcome measure

The outcome is the identified potential differences deep learning features between GC without PM group and GC with PM group will be the outcome measures.

### 2.8 Data collection and management

Data will be processed anonymously, omitting the information that can identify the participant's individual identity. Strict safety and confidentiality measures will be established in the archives of clinical trial institutions.

### 2.9 Sample size calculation

We calculated the number of patients needed considering the epidemiology of PM in GC; the estimated sample size was at least 109.

### 2.10 Statistical analysis

The differences of continuous variables were analyzed by the Mann–Whitney *U* test, and the differences of categorical variables were analyzed by the Chi-square test. The accuracy of the deep learning-based model was assessed with the receiver operating characteristic (ROC) curve and area under the ROC curve. Delong test was used to compare the ROC curves of models. The calibration of the model was assessed using the calibration curves. In addition, sensitivity and specificity for classification were evaluated.

For the deep learning-based model, 2 different probability values were selected as classification thresholds based on the results of the in-house validation cohort: a high sensitivity threshold (75% sensitivity on in-house validation) and a high specificity threshold (75% specificity on in-house validation). Considering the group imbalance in the in-house validation cohort, we used bootstrap (resampled the in-house validation cohort 1000 times) and calculated the 95% confidence interval. Finally, the decision curve was conducted to evaluate our approach usefulness by quantifying the net benefits at different thresholds probabilities.

All these statistical analyses were performed with SPSS 22.0. A 2-tailed *P*-value less than .05 was considered statistically significant.

### 2.11 Ethics and dissemination

This retrospective study was approved by the Biomedical Research Ethics Committee of West China Hospital of Sichuan University (No. 2019-1158), and the requirement for informed consent was waived. To protect privacy of participants, all of private information were anonymous. The results will be published in a peer-reviewed journal and will be disseminated electronically and in print regardless of results. The authors report no conflicts of interest.

## 3 Discussion

In this study, we will develop a deep learning-based model to preoperative identify occult PM in gastric cancer (AGC). The model may provide a preoperative, and easy-to-use tool for occult PM diagnosis, which can help avoid improper clinical management for patients with occult PM or determine optimal candidates for laparoscopy exploration.

A lot of imaging studies have focused on preoperative assessment of the PM status in GCs.^[11,15–17,28]^ CT should be the preferred diagnostic imaging modality for detecting PM because of the robustness of the data, while MRI and positron emission tomography-computed tomography (PET/CT) should be considered second choices, until more consistent information on their diagnostic yield in detecting PM are obtained.^[29]^ However, diagnostic performance of CT varies substantially among previously reported studies and its overall sensitivity is poor.^[30]^ Recently, a few CT radiomics studies focused on PM of GC. Dong et al reported that venous CT radiomics analysis combining both primary tumor and nearby peritoneum had an excellent prediction value of occult PM of AGC.^[24]^ In another study, Liu et al found that venous CT radiomics analysis based on the primary tumor provided valuable information for predicting occult PM in AGC.^[23]^ The repeatability of radiomics results is easily affected by different image acquisition and reconstruction protocols,^[31]^ while deep learning can avoid these disadvantages of radiomics.^[32]^ The classic theory of tumor metastasis is the “seed and soil” theory, which is now widely accepted,^[33]^ and we also agree with it. But, we did not delineate the peritoneal ROIs as Dong et al^[24]^ did, we were skeptical whether a small 2D area of peritoneum near the tumor would represent the “soil” condition. We are more willing to construct a relationship with PM from the characteristics of the primary tumor.

Our study has several limitations. The ROIs were delineated in 1 single slice (2D), which might not be representative of the entire tumor. Meanwhile, development of model may be affected some input of model extracted from 2D versus 3D images. Therefore, 3D analysis of the entire tumor should be further investigated. Moreover, we used the retrospective datasets to develop deep learning model, of which some clinical factors such as serological tumor markers were not initially available on account of incomplete data.

We hope that the deep learning-based model can be an excellent prediction ability of occult PM and may have significant clinical implications on early detection of occult PM for AGC.

## Author contributions

**Conceptualization:** Zixing Huang, Bing Wu.

**Data curation:** Dan Liu, Xinzu Chen.

**Deep learning method:** Pengxin Yu, Jiangfen Wu.

**Methodology:** Zixing Huang, Pengxin Yu.

**Resources:** Bin Song, Jiankun Hu.

**Supervision:** Bin Song, Jiankun Hu, Bing Wu.

**Writing – original draft:** Zixing Huang, Dan Liu, Xinzu Chen.

**Writing – review and editing:** Zixing Huang, Bin Song, Jiankun Hu, Bing Wu.
